# Recognition properties of receptors consisting of imidazole and indole recognition units towards carbohydrates

**DOI:** 10.3762/bjoc.6.9

**Published:** 2010-02-02

**Authors:** Monika Mazik, André Hartmann

**Affiliations:** 1 Institut für Organische Chemie der Technischen Universität Braunschweig, Hagenring 30, 38106 Braunschweig, Germany, Tel.: +495313915266, Fax: +495313918185

**Keywords:** carbohydrates, hydrogen bonds, molecular recognition, receptors, supramolecular chemistry

## Abstract

Compounds **4** and **5**, including both 4(5)-substituted imidazole or 3-substituted indole units as the entities used in nature, and 2-aminopyridine group as a heterocyclic analogue of the asparagine/glutamine primary amide side chain, were prepared and their binding properties towards carbohydrates were studied. The design of these receptors was inspired by the binding motifs observed in the crystal structures of protein–carbohydrate complexes. ^1^H NMR spectroscopic titrations in competitive and non-competitive media as well as binding studies in two-phase systems, such as dissolution of solid carbohydrates in apolar media, revealed both highly effective recognition of neutral carbohydrates and interesting binding preferences of these acyclic compounds. Compared to the previously described acyclic receptors, compounds **4** and **5** showed significantly increased binding affinity towards β-galactoside. Both receptors display high β- vs. α-anomer binding preferences in the recognition of glycosides. It has been shown that both hydrogen bonding and interactions of the carbohydrate CH units with the aromatic rings of the receptors contribute to the stabilization of the receptor–carbohydrate complexes. The molecular modeling calculations, synthesis and binding properties of **4** and **5** towards selected carbohydrates are described and compared with those of the previously described receptors.

## Introduction

**Figure 1:**
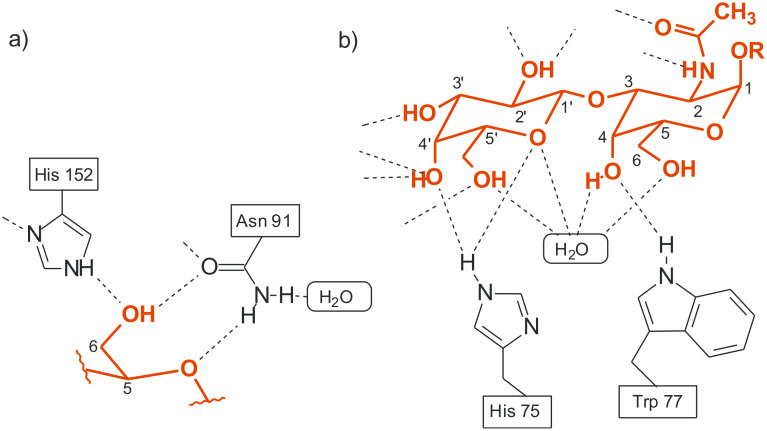
Examples of hydrogen bonds in the complex of a) galactose-binding protein with D-glucose [3], b) *Amaranthus caudatus* agglutinin with Galβ3GalNAc [1].

**Figure 2:**
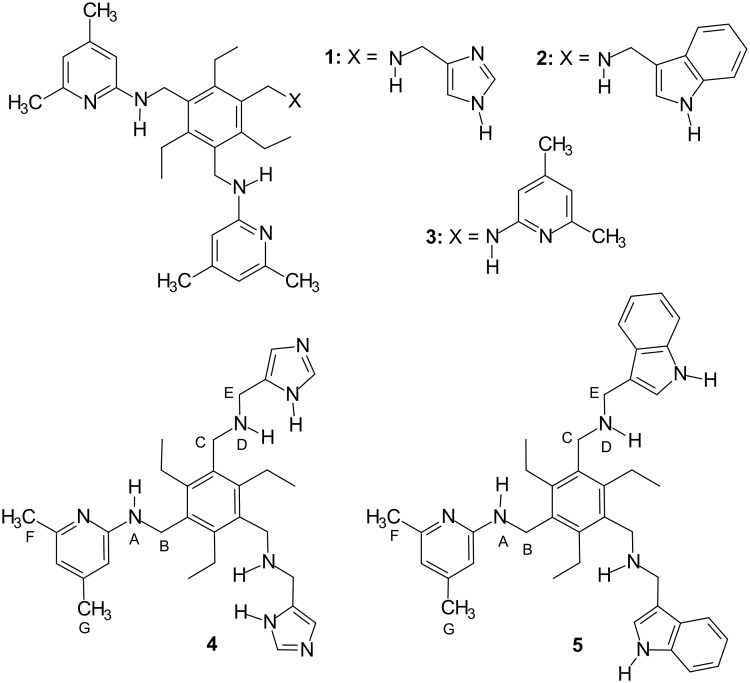
Structures of receptors **1**–**5**.

Analysis of the binding motifs found in the crystal structures of protein–carbohydrate complexes [1–5] provides much of the inspiration for the design of artificial carbohydrate receptors which use noncovalent interactions for sugar binding [6–18]. Such receptors provide valuable model systems to study the underlying principles of carbohydrate-based molecular recognition processes and might serve as a basis for the development of new therapeutic agents (for example, anti-infective agents) or saccharide sensors [19–26]. Our previous studies showed that mimicking the binding motifs observed in the crystal structures of protein–carbohydrate complexes by using natural recognition groups or their analogues [27–45] represents an effective strategy for designing carbohydrate receptors. Among other things the crystal structures of protein–carbohydrate complexes revealed that the imidazole and indole groups of His and Trp respectively are able to participate in both hydrogen bonding and stacking interactions with the sugar ring. It should be noted that packing of an aromatic ring of the protein against a sugar is observed in most carbohydrate–binding proteins [1–5]. Such packing arrangements and the hydrogen bonding motifs shown in Figure 1 have inspired the design of receptors **1** and **2** (see Figure 2), including both 4(5)-substituted imidazole or 3-substituted indole units as the entities used in nature, and 2-aminopyridine groups as heterocyclic analogues of the asparagine/glutamine primary amide side chains (in analogy to the binding motif shown in Figure 1a) [31]. The compounds **1** and **2** were established as highly effective receptors for mono- and disaccharides and shown to display remarkable β- vs. α-anomer selectivity in the recognition of glucopyranosides, as well as a binding preference for β-glucopyranoside vs. β-galactopyranoside. It has been shown that both hydrogen bonding and interactions of the carbohydrate CH units with the aromatic rings of the receptors contribute to the stabilization of the receptor–carbohydrate complexes. Compounds **1** and **2** were shown to be more powerful carbohydrate receptors than the symmetrical aminopyridine-based receptor **3**.

**Figure 3:**
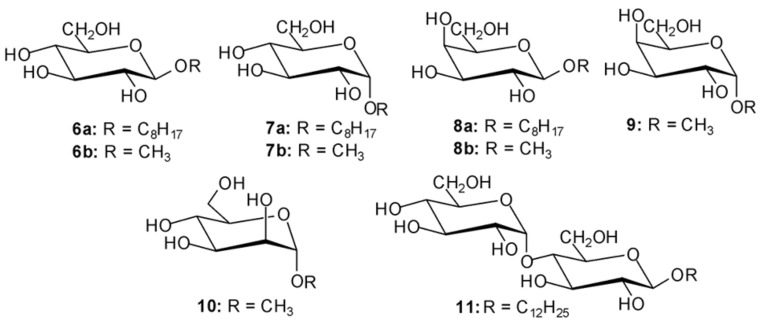
Structures of sugars investigated in this study.

We were interested to see whether compounds **4** and **5** (see Figure 2), which consist of two imidazole or indole groups and one 2-aminopyridine unit, would be more effective with mono- and disaccharide substrates. Herein, we describe the synthesis, molecular modeling calculations and the binding properties of the compounds **4** and **5**. To compare the binding properties of the new compounds with those of the previously published receptors, octyl β-D-glucopyranoside (**6a**), methyl β-D-glucopyranoside (**6b**), octyl α-D-glucopyranoside (**7a**), methyl α-D-glucopyranoside (**7b**), octyl β-D-galactopyranoside (**8a**), methyl β-D-galactopyranoside (**8b**), methyl α-D-galactopyranoside (**9**), methyl α-D-mannopyranoside (**10**) and dodecyl β-D-maltoside (**11**) were selected as substrates for the binding experiments (see Figure 3). ^1^H NMR spectroscopic titrations in competitive and non-competitive media as well as binding studies in two-phase systems, such as dissolution of solid carbohydrates in apolar media, revealed highly effective recognition of neutral carbohydrates and interesting binding preferences of these acyclic receptors.

## Results and Discussion

### Synthesis of the receptors

**Scheme 1:**
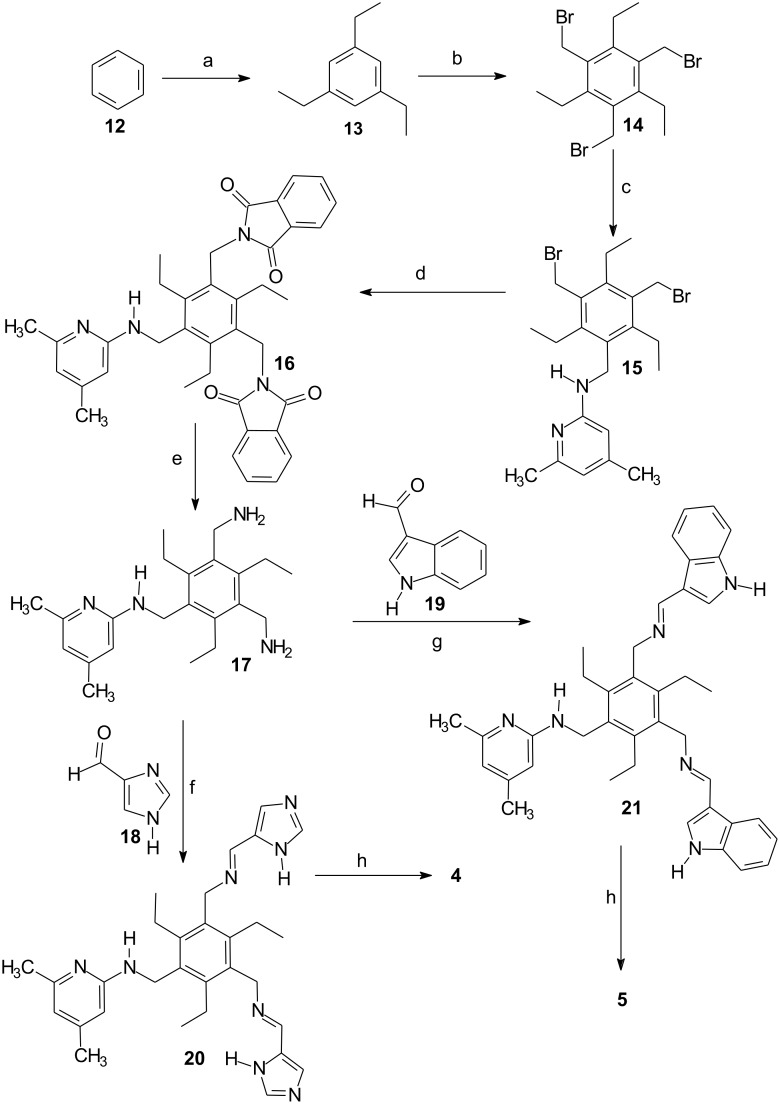
Reaction conditions: a) AlCl_3_, CH_3_CH_2_Br, 0 °C to r. t., 12 h (85%) [47]; b) 33% HBr in CH_3_COOH, ZnBr_2_, (CH_2_O)*_n_*, 90 °C, 16.5 h (94%); c) 2 equiv of 2-amino-4,6-dimethylpyridine, CH_3_CN/THF, K_2_CO_3_, r. t., 3 d (20%); d) potassium phthalimide, dimethyl sulfoxide, 95 °C, 8 h, (57%); e) hydrazine hydrate, ethanol/toluene, reflux, 19.5 h, KOH (43%) [27]; f) 4 equiv of 4(5)-imidazole-carbaldehyde (**18**), CH_3_OH, 3 d; g) 4 equiv of 3-indole-carbaldehyde (**19**) CH_3_OH, 3 d; h) 8 equiv of NaBH_4_, 0 °C to r. t., 12 h (78% of **4**, 92% of **5**).

The basis for the synthesis of compounds **4** and **5** was 1,3-bis(aminomethyl)-5-[(4,6-dimethylpyridin-2-yl)aminomethyl]-2,4,6- triethylbenzene (**17**). The synthesis of compound **17** is described in reference [27]. The reaction of **17** with the corresponding carbaldehyde, such as 4(5)-imidazole-carbaldehyde (**18**) [46] or 3-indole-carbaldehyde (**19**), provided the corresponding imines **20** and **21**, which were further reduced with sodium borohydride. The synthesis of receptors **4** and **5** is summarized in Scheme 1.

### Binding studies in two-phase systems: liquid-solid extractions

**Figure 4:**
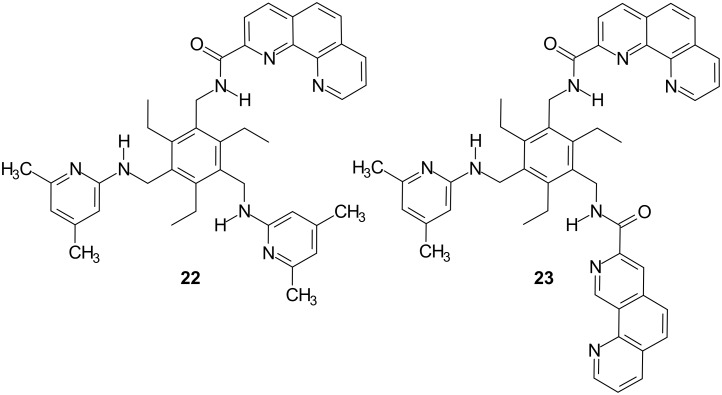
Structures of the recently described phenanthroline/aminopyridine-based receptors showing α- vs. β-anomer binding preferences in the recognition of glycosides [27,29].

The dissolution of solid carbohydrates in apolar media provides valuable means of studying carbohydrate recognition by organic-soluble receptors (for examples of receptors which are able to dissolve solid carbohydrates in apolar media, see references [6,27,41,43,48–50]). Extractions of sugars **6b**, **7b**, **8b**, **9** and **10** from the solid state into a CDCl_3_ solution of receptor **4** or **5** (1 mM) provided evidence for strong complexation of β-glucoside **6b** and β-galactoside **8b**. The extraction of solid methyl α-glucoside **7b**, α-galactoside **9** and α-mannoside **10** into a CDCl_3_ solution of receptor **4** or **5** indicated a weaker binding of these sugars than that of **6b** and **8b** (see Table 1). The extraction experiments indicated that the imidazole-based receptor **4** is a more powerful carbohydrate receptor than the indole-based compound **5**. Receptor **4** was able to dissolve about 1 equiv of β-glucoside **6b** and β-galactoside **8b**, 0.5 equiv of α-glucoside **7b** and about 0.2 equiv of α-galactoside **9**. In the case of receptor **5** only about 0.7 equiv of β-glucoside **6b** and β-galactoside **8b** could be detected in the solution (see Table 1). Regarding **4** and **5**, the extractability decreased in the sequence β-glucoside **6b** ~ β-galactoside **8b** > α-glucoside **7b** > α-galactoside **9** > α-mannoside **10** (see Table 1; control experiments were performed in the absence of the receptor). The preference of **4** and **5** for β- vs. α-glucoside (**6b** vs. **7b**) as well as for β- vs. α-galactoside (**8b** vs. **9**) indicated by liquid–solid extractions was further confirmed by ^1^H NMR spectroscopic titrations (see below). Compared to the previously studied receptors **1**–**3**, the extraction experiments indicated a significantly higher level of affinity of **4** and **5** towards β-galactoside. It should also be noted that the selectivities observed for **4** and **5** are quite different to those of the recently described phenanthroline/aminopyridine-based receptors **22** and **23** (see Figure 4) [27,29], which show a strong preference for α-glucoside and α-galactoside vs. the β-anomers. Thus, depending on the nature of the recognition units used as building blocks for the acyclic structures, effective carbohydrate receptors with different binding selectivities could be obtained. However, the exact prediction of the binding selectivity still represents an unsolved problem.

**Table 1:** Solubilization of sugars in CDCl_3_ by receptor **4** and **5** (1 mM solution).

Sugar	Sugar/**4**^a^	Sugar/**5**^a^

β-D-glucoside **6b**	0.98	0.72
α-D-glucoside **7b**	0.50	0.19
β-D-galactoside **8b**	0.95	0.74
α-D-galactoside **9**	0.20	0.09
α-D-mannoside **10**	0.11	0.04

^a^Molar ratios sugar/receptor occurring in solution (the ^1^H NMR signals of the corresponding sugar were integrated with respect to the receptor’s signals to provide the sugar–receptor ratio; control experiments were performed in the absence of the receptor).

### Binding studies in homogeneous solution

The interactions of the receptors and carbohydrates were investigated by ^1^H NMR spectroscopic titrations in CDCl_3_ and DMSO-*d*_6_/CDCl_3_ mixtures. The stoichiometry of the receptor–sugar complexes was determined by mole ratio plots [51–52] and by the curve-fitting analysis of the titration data [53].

**Figure 5:**
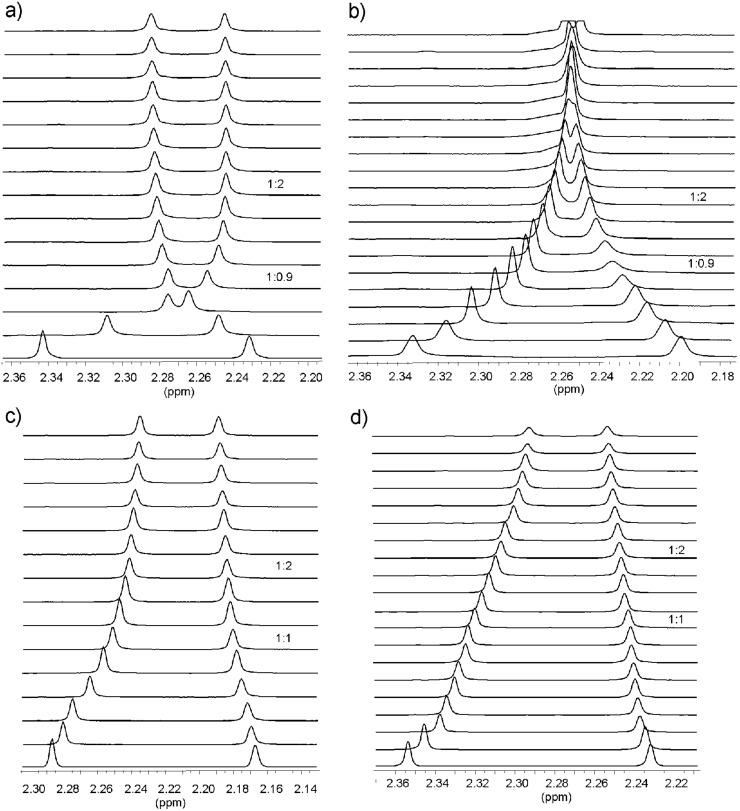
Partial ^1^H NMR spectra (400 MHz; CDCl_3_) of receptor **4** (a), **5** (b), **1** (c), and **3** (d) before (bottom) and after the addition of β-galactoside **8a**. Shown are chemical shifts of the pyridine CH_3_ resonances of the corresponding receptor. [**4**] = 0.89 mM, equiv of **8a**: 0.00–4.65; [**5**] = 0.90 mM, equiv of **8a**: 00–4.52; [**1**] = 0.95 mM, equiv of **8a**: 0.00–4.26; [**3**] = 0.90 mM, equiv of **8a**: 0.00–5.20.

The ^1^H NMR titration experiments [54] with octyl β-glucoside **6a**, α-glucoside **7a**, β-galactoside **8a** and methyl α-galactoside **9** were carried out by adding increasing amounts of sugar to a solution of receptor **4** or **5**. In addition, inverse titrations were performed in which the concentration of the sugar was held constant and that of the receptor was varied. The complexation between receptors **4** or **5** and the monosaccharides was evidenced by several changes in the NMR spectra (for examples, see Table 2 and Figure 5a and Figure 5b). The addition of the monosaccharides **6a**, **7a** or **8a** to a CDCl_3_ solution of receptors **4** or **5** caused significant downfield shift of the amine NH^A^ signal (for labeling, see Figure 2), downfield shift and strong broadening of the NH^D^ signal as well as changes of the chemical shifts of the CH_3_^F,G^, CH_2_^B,C,E^, pyridine CH and imidazole or indole CH resonances of **4** or **5** (see Table 2). The signal due to the indole NH of **5** shifted downfield by 0.20–0.40 ppm. The complexation-induced chemical shifts of the NH^A^, indole-NH, CH_2_^B^, CH_3_^F,G^ and the aromatic CH protons were monitored for the determination of the binding constants, which are summarized in Table 3. Binding studies with β-glucoside **6a** and β-galactoside **8a** showed the interactions of receptors **4** and **5** with these monosaccharides to be much more favorable than those with the α-anomers **7a** and **9**.

**Table 2:** Change in chemical shift^a^ observed during ^1^H NMR titrations of receptor **4** or **5** with sugar **6a**, **7a**, **8a** or **11** in CDCl_3_.

Receptor– sugar complex	Δδ^a^ [ppm]

**4**•**6a**	NH^A^: 2.01; CH_2_^B^: −0.17; imidazole-CH’s: 0.06, 0.08; CH_3_^F^: −0.07
**4**•**7a**	NH^A^: 1.17; CH_2_^B^: −0.15; imidazole-CH’s: 0.05, 0.06; CH_3_^F^: −0.05
**4**•**8a**	NH^A^: 0.79; CH_2_^B^: −0.12; CH_2_^E^: −0.11; imidazole-CH’s: 0.11, 0.08; CH_3_^F^: −0.10; CH_3_^G^: 0.05
**4**•**11**	NH^A^: 0.80; CH_2_^B^: −0.19; CH_2_^C^: −0.09; imidazole-CH’s: 0.09, 0.04; CH_3_^F^: −0.06; CH_3_^G^: 0.03
**5**•**6a**	NH^A^: 2.06; indole-NH: 0.20; CH_2_^B^: −0.18; CH_2_^E^: −0.06; CH_3_^F^: −0.07; CH_3_^G^: 0.04
**5**•**7a**	NH^A^: 1.50; indole-NH: 0.17; CH_2_^B^: −0.18; CH_2_^C^: −0.06; CH_3_^F^: −0.06
**5**•**8a**	NH^A^: 1.15; indole-NH: 0.27; CH_2_^B^: −0.15; CH_2_^C^: 0.06; CH_2_^E^: −0.11; pyr-CH’s: −0.01, 0.11; CH_3_^F^: −0.09; CH_3_^G^: 0.06
**5**•**11**	NH^A^: 1.80; indole-NH: 0.40; CH_2_^B^: −0.20; CH_3_^F^: −0.06; CH_3_^G^: 0.04

^a^Largest change in chemical shift observed during the titration for receptor signals (the concentration of receptor was kept constant and that of sugar varied). ^b^(−) Δδ = upfield shift.

**Table 3:** Association constants^a,b^ for receptors **1**–**6** and carbohydrates **6a**, **7a**, **8a**, **9** and **11**.

Host–guest complex	Solvent	*K*_11_ [M ^−1^]	*K*_21_^c^ or *K*_12_^d^ [M^−1^]	β_21_ = *K*_11_*K*_21_ or β_12_ = *K*_11_*K*_12_ [M^−2^]

**4**•**6a**	CDCl_3_	>10^5^; ^g^	^g^	
	5% DMSO-*d*_6_/CDCl_3_	35000	1000; ^d^	3.50×10^7^
**4**•**7a**	CDCl_3_	7450	1150; ^d^	8.56×10^6^
**4**•**8a**	CDCl_3_	>10^5^; ^g^	^g^	
	5% DMSO-*d*_6_/CDCl_3_	40700	800; ^d^	3.25×10^7^
**4**•**9**	5% DMSO-*d*_6_/CDCl_3_	700		
**4**•**11**	CDCl_3_	>10^5^; ^g^	^g^	
	5% DMSO-*d*_6_/CDCl_3_	12000	3000; ^c^	3.60×10^7^
				
**5•6a**	CDCl_3_	45900	730; ^d^	3.35×10^7^
**5**•**7a**	CDCl_3_	1280	250; ^d^	3.20×10^5^
**5**•**8a**	CDCl_3_	38000	1100; ^d^	4.18×10^7^
**5**•**11**	CDCl_3_	>10^5^; ^g^	^g^	
	5% DMSO-*d*_6_/CDCl_3_	42000		
**1**•**6a**^e^	CDCl_3_	191730	8560; ^c^	1.64×10^9^
**1**•**7a**^e^	CDCl_3_	3160	1540; ^d^	4.86×10^6^
**1**•**8a**^e^	CDCl_3_	3320	300; ^d^	9.96×10^5^
**1**•**11**^e^	CDCl_3_	205760	8670; ^c^	1.78×10^9^
				
**2**•**6a**^e^	CDCl_3_	156100	10360; ^c^	1.62×10^9^
**2**•**7a**^e^	CDCl_3_	2820	350; ^d^	9.87×10^5^
**2**•**8a**^e^	CDCl_3_	7470	1100; ^d^	8.25×10^6^
**2**•**11**^e^	CDCl_3_	182690	14840; ^c^	2.71×10^9^
				
**3**•**6a**^f^	CDCl_3_	48630	1320; ^d^	6.42×10^7^
**3**•**7a**^f^	CDCl_3_	1310		
**3•8a**^f^	CDCl_3_	3070	470; ^d^	1.35×10^6^

^a^Average *K*_a_ values from multiple titrations in CDCl_3_. ^b^Errors in *K*_a_ are less than 10%. ^c^*K*_21_ corresponds to 2:1 receptor–sugar association constant. ^d^*K*_12_ corresponds to 1:2 receptor–sugar association constant. ^e^Results from ref. [31]. ^f^Results from ref. [41]. ^g^Hostest program indicated “mixed” 1:1 and 2:1 receptor-sugar binding model with *K*_11_>10^5^ and *K*_21_ ~ 10^4^; however, the binding constants were too large to be accurately determined by the NMR method.

The curve fitting of the titration data for **4** and β-glucoside **6a** suggested the existence of 1:1 and 2:1 receptor–sugar complexes in CDCl_3_ solutions with a stronger association constant for 1:1 binding and a weaker association constant for the 2:1 receptor–sugar complex (this model was further supported by the mole ratio plots). The binding constants, however, were too large to be accurately determined by the NMR spectroscopic method (*K*_11_ > 10^5^ and *K*_21_ ~ 10^4^ M^−1^; see Table 3; for a review discussing the limitations of the NMR method, see ref. [55]). After the addition of 5% DMSO-*d*_6_ the binding constants for **4**•**6a** were determined to be 35000 (*K*_11_) and 1000 M^−1^ (*K*_12_). Thus, the affinity of **4** significantly decreases as solvent polarity increases (the addition of dimethyl sulfoxide also caused the change of the binding model; for a discussion on solvent effects in carbohydrate binding by synthetic receptors, see ref. [56]).

The interactions between the β-glucoside **6a** and the indole-based receptor **5** in CDCl_3_ were shown to be strong but less favorable than those with the receptor **4**. The best fit of the titration data was obtained with the “mixed” 1:1 and 1:2 receptor–sugar binding model. The association constants for **5**•**6a** were found to be 45900 (*K*_11_) and 730 M^−1^ (*K*_12_).

The interactions between β-glucopyranoside **6a** and receptors **4** and **5** were also investigated on the basis of inverse titrations in which the concentration of sugar **6a** was held constant and that of receptor **4** or **5** was varied. During the titration of **6a** with **4** or **5** the signals due to the OH protons of **6a** shifted downfield with strong broadening and became almost indistinguishable from the base line after the addition of only 0.1 equiv of the receptor, indicating important contribution of the OH groups of **6a** to the complex formation. Furthermore, the addition of **4** or **5** to a CDCl_3_ solution of β-glucoside **6a** caused significant upfield shift of the CH signals of **6a**, indicating the participation of the sugar CH units in the formation of the CH···π interactions with the aromatic rings of the receptor (for discussions on the importance of carbohydrate–aromatic interactions, see refs. [57–63]; for examples of CH-π interactions in the crystal structures of the complexes formed between artificial receptors and carbohydrates, see ref. [40]). Among the CH signals, the signal due to the 2-CH proton of **6a** showed the largest shift (1.78 and 1.62 ppm for the titration with **4** and **5**, respectively). In both cases, **6a**•**4** and **6a**•**5**, the best fit of the titration data was obtained with the “mixed” 1:1 and 1:2 sugar–receptor binding model. Thus, the inverse titrations fully confirmed the binding model determined through the titrations of **4** or **5** with sugar **6a**. The association constants obtained on the basis of these titrations are identical within the limits of uncertainty to those determined from titrations where the role of receptor and substrate was reversed.

Similar to **4**•**6a**, the best fit of the titration data for receptor **4** and β-galactoside **8a** was obtained with the “mixed” 1:1 and 2:1 receptor–sugar binding model. However, the binding constants were again too large to be accurately determined by the NMR spectroscopic method (see Table 3). Studies performed in 5% DMSO-*d*_6_ in CDCl_3_ revealed that *K*_11_ = 40700 M^−1^ and *K*_12_ = 800 M^−1^. The titration experiments with β-galactoside **8a** clearly showed that receptor **5** is less effective towards this monosaccharide than the imidazole-based receptor **4** but much more effective than the previously described receptors **1**–**3**. The motions of the signals of **5** were consistent with 1:1 and 1:2 receptor–sugar binding and could be analyzed to give association constants of 38000 (*K*_11_) and 1100 M^−1^ (*K*_12_). Compared to receptors **1**–**3** [31,41], receptors **4** and **5** showed a significantly higher binding affinity towards the β-galactoside **8a**. The differences in the complexation abilities of receptors **1**/**3** and **4**/**5** towards β-galactoside **8a** are clearly visible in the comparison of the chemical shifts of the signals of the four receptors after the addition of β-galactoside **8a** (illustrated in parts a–d of Figure 5 for the pyridine CH_3_ signals).

**Figure 6:**
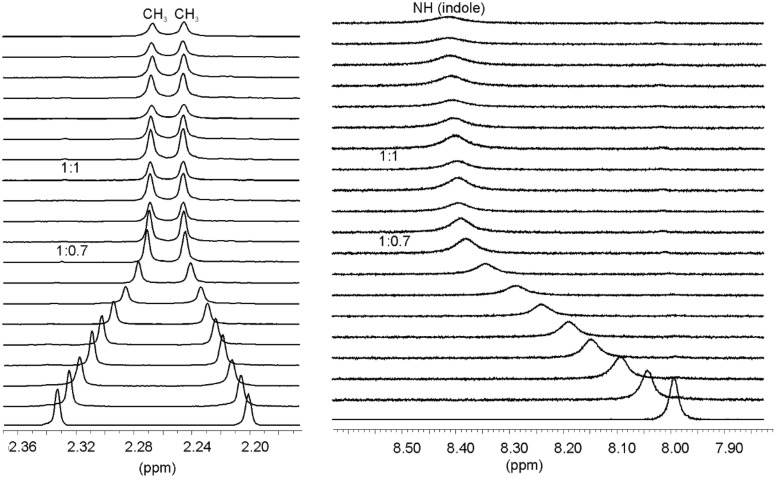
Partial ^1^H NMR spectra (400 MHz, CDCl_3_) of **5** after addition of (from bottom to top) 0.00–1.63 equiv of β-maltoside **11** ([**5**] = 0.96 mM). Shown are chemical shifts of the pyridine CH_3_ and indole NH signals of receptor **5**.

Our previous studies showed compounds **1**–**3** to be highly effective receptors for β-maltoside **11** [28,31]. This disaccharide [64] is almost insoluble in CDCl_3_ but could be solubilized in this solvent in the presence of the corresponding receptor. Similar solubility behavior of **11**, indicating favorable interactions between the binding partners, could be observed in the presence of compounds **4** and **5**. Thus, the receptor in CDCl_3_ was titrated with a solution of maltoside dissolved in the same receptor solution. The complexation between **4** or **5** and the disaccharide **11** was evidenced by several changes in the NMR spectra (for example, see Table 2 and Figure 6). The saturation occurred after the addition of about 0.7 equiv of **11**. Both the curve fitting of the titration data and and the mole ratio plots suggested the existence of 1:1 and 2:1 receptor–sugar complexes in the chloroform solution (with stronger association constant for 1:1 binding and a weaker association constant for 2:1 receptor–sugar complex). In both cases, **4**•**11** and **5**•**11**, the binding constants in CDCl_3_ were too large to be accurately determined by the NMR spectroscopic method (see Table 3). After the addition of DMSO-*d*_6_ a substantial fall in the binding affinity was observed. Studies that were performed with **4** and **11** in 5% DMSO-*d*_6_ in CDCl_3_ revealed *K*_11_ = 12000 M^–1^ and *K*_21_ = 3000 M^–1^, those performed with **5** and **11** indicated the formation of complexes with 1:1 receptor–sugar stoichiometry with *K*_11_ = 42000 M^–1^.

### Molecular modeling

**Figure 7:**
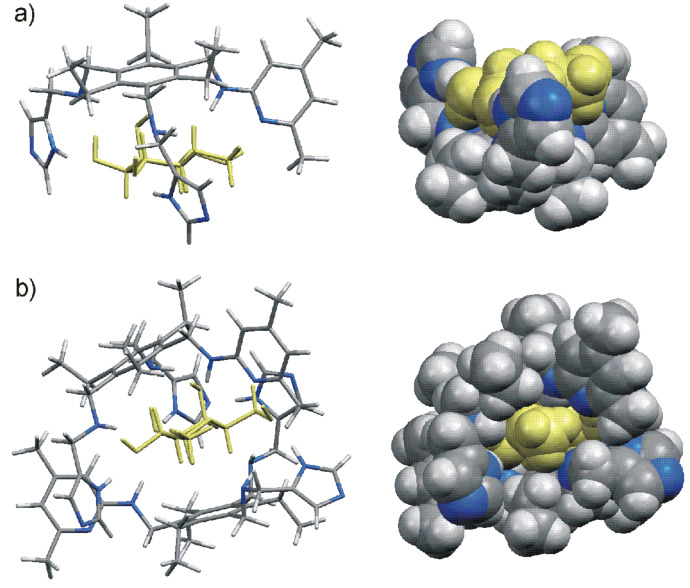
Energy-minimized structure of the 1:1 a) and 2:1 complex b) formed between receptor **4** and β-galactoside **8b** (different representations). MacroModel V.8.5, OPLS-AA force field, MCMM, 50000 steps. Color code: receptor C, grey; receptor N, blue; sugar molecule, yellow.

**Figure 8:**
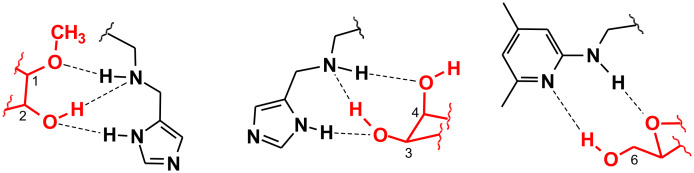
Examples of hydrogen bonding motifs indicated by molecular modeling studies in the 1:1 complex between receptor **4** and β-galactoside **8b** (MacroModel V.8.5, OPLS-AA force field, MCMM, 50000 steps).

The formation of hydrogen bonds and CH···π interactions between the binding partners was also suggested by molecular modeling calculations. For example, molecular modeling suggested that all OH groups and the ring oxygen atom of the bound β-galactoside **8b** in the complex **4**•**8b** are involved in the formation of hydrogen bonds (see Table 4 and Figure 7a and Figure 8). In addition, interactions of sugar C-H units with the central phenyl ring of **4** (see Table 4) were shown to provide additional stabilization of the complex. Furthermore, the molecular modeling calculations indicated that within the 2:1 receptor–sugar complex the two receptor molecules almost completely enclose the sugar, leading to involvement of all sugar hydroxyl groups in interactions with the two receptor molecules (see Table 4 and Figure 7b). The OH groups are involved in the formation of cooperative hydrogen bonds which result from the simultaneous participation of a sugar OH as donor and acceptor of hydrogen bonds. The phenyl units of the both receptors stack on the sugar ring and both sides of the pyranose ring are involved in CH···π interactions (see Table 4 and Figure 7b).

**Table 4:** Examples of noncovalent interactions indicated by molecular modeling calculations^a^ for the complexes formed between receptor **4** and sugar **8a** or **8b**.

1:1 receptor–sugar complex^b^	2:1 receptor–sugar complex^b,c^	1:2 receptor–sugar complex^d^

imidazole-NH···OH-2	(I) imidazole-NH···OH-2	imidazole-NH···OH-6
HN^D^···HO-2	(I) HN^D^···HO-2	HN^D^···HO-6
NH^D^···O-CH_3_	(I) NH^D^···O-CH_3_	NH^D^···OH-4
imidazole-NH···OH-3	(I) imidazole-NH···OH-3	imidazole-NH···OH-4
HN^D^···HO-3	(I) HN^D^···HO-3	pyridine-N···HO-2
NH^D^···OH-4	(I) NH^D^···OH-4	NH^A^···OC_8_H_17_
phenyl···HO-4	(I) pyridine-N···HO-6	phenyl···HO-4
pyridine-N···HO-6	(I) NH^A^···O-ring	phenyl···HCH-6
NH^A^···O-ring	(I) phenyl···HO-4; (I) phenyl···HC-2	pyridine-N···HC-2^e^
phenyl···HC-2	(II) imidazole-NH···OH-6; (II) NH^D^···OH-6	pyridine-CH_3_···OH-4^e^
	(II) NH^A^···OH-3	3-HO···HO-2^f^
	(II) phenyl···HC-1	3-OH···OH-3^f^
	(II) phenyl···HC-3; (II) phenyl···HC-5	

^a^MacroModel V.8.5, OPLS-AA force field, MCMM, 50000 steps. ^b^Complex with sugar **8b**. ^c^I and II: two receptors in the 2:1 receptor–sugar complex; for labeling see Figure 2. ^d^Complex with sugar **8a**. ^e^Interaction with the second sugar. ^f^Sugar–sugar interaction.

## Conclusion

The analysis of the binding motifs which are observed in the crystal structures of protein-carbohydrate complexes has influenced the design of receptors **4** and **5**, including two 4(5)-substituted imidazole or 3-substituted indole units as well as an aminopyridine-based recognition group. The compounds **4** and **5** were established as highly effective receptors for neutral carbohydrates and were shown to display a significantly higher level of affinity towards β-galactoside than the previously described acyclic receptors. Both receptors were shown to display high β- vs. α-anomer binding preferences in the recognition of glycosides. The binding properties of **4** and **5** were studied on the base of ^1^H NMR spectroscopic titrations in CDCl_3_ and DMSO-*d*_6_/CDCl_3_ mixtures as well as binding studies in two-phase systems, such as dissolution of solid carbohydrates in apolar media. The imidazole-based receptor **4** was found to be a more powerful monosaccharide receptor than the indole-based compound **5** and the previously described receptors **1**–**3**. Compared to **1** and **2**, incorporating only one imidazole or indole recognition unit, receptor **5** showed increased affinity to β-galactoside but decreased affinity to β-glucoside. The binding affinity of **1**–**5** towards β-galactoside **8a** and β-glucoside **6a** increases in the sequence **3** ~ **1** < **2** < **5** < **4** and **3** ~ **5** < **1** ~ **2** < **4**, respectively. It is remarkable that the strong enhancement of the binding affinity of **4** and **5** towards β-galactoside was achieved through a relatively simple variation of the receptor structure. In contrast to **4** and **5**, the previously described phenanthroline/aminopyridine-based receptors **22** and **23** were shown to display a high binding affinity towards α-galactoside as well as a strong α- vs. β-anomer binding preference. Thus, depending on the nature of the recognition units incorporated into the acyclic receptor structure, effective carbohydrate receptors with different binding preferences can be generated. However, the exact prediction of the binding preference still represents an unsolved problem and remains an important goal for future research.

## Experimental section

Analytical TLC was carried out on silica gel 60 F_254_ plates employing chloroform/methanol mixtures as the mobile phase. Melting points are uncorrected. Sugars **6**–**11**, 4(5)-imidazole-carbaldehyde (**18**) and 3-indole-carbaldehyde (**19**) are commercially available.

**General procedure for the synthesis of compounds 4 and 5:** To a solution of 4(5)-imidazole-carbaldehyde (**18**) or 3-indole-carbaldehyde (**19**) (3.40 mmol) in methanol (40 mL) 1,3-bis(aminomethyl)-5-[(4,6-dimethylpyridin-2-yl)aminomethyl]-2,4,6-triethylbenzene (**17**) (0.85 mmol) dissolved in 20 mL methanol was added. The reaction mixture was stirred for 72 h. The solution was cooled to 0 °C and NaBH_4_ (6.80 mmol) was added in portions. The reaction mixture was stirred for 1 h at 0 °C and for additionally 6 h at room temperature. The solvent was removed and the residue was taken up in chloroform/water (100 mL, 1:1). The separated organic phase was further washed with water (3×30 mL), dried over MgSO_4_ and the solvent was removed. The crude product was purified via column chromatography [CHCl_3_/CH_3_OH (incl. 1% 7 M NH_3_ in CH_3_OH), 2:1 or 3:1 *v*/*v*].

**1,3-Bis[(4-Imidazolyl-methyl)aminomethyl]-5-[(4,6-dimethylpyridin-2-yl)aminomethyl]-2,4,6-triethylbenzene (4).** Yield: 78%; mp: 76–77 °C; ^1^H NMR (400 MHz, CDCl_3_, 0.9 mM): δ = 7.54 (s, 2H), 6.93 (s, 2H), 6.33 (s, 1H), 6.07 (s, 1H), 4.28 (s, 2H), 4.18 (br. s, 1H), 3.89 (s, 4H), 3.71 (s, 4H), 2.68 (q, *J* = 7.3 Hz, 4H), 2.65 (q, *J* = 7.3 Hz, 2H), 2.34 (s, 3H), 2.23 (s, 3H), 1.17 (t, *J* = 7.3 Hz, 6H), 1.09 (t, *J* = 7.3 Hz, 3H) ppm; ^13^C NMR (100 MHz, CDCl_3_): δ = 158.33, 156.44, 148.96, 142.75, 142.43, 135.65, 134.07, 132.41, 113.85, 103.58, 46.75, 46.05, 24.01, 22.70, 22.50, 21.11, 16.83, 16.80 ppm; HR-MS (ESI) calcd for C_30_H_42_N_8_Na [M + Na]^+^: 537.3430, found: 537.3433; *R_f_* = 0.10 [CHCl_3_/CH_3_OH (incl. 1% 7 M NH_3_ in CH_3_OH), 4:1 *v*/*v*].

**1,3-Bis[(3-Indolyl-methyl)aminomethyl]-5-[(4,6-dimethylpyridin-2-yl)aminomethyl]-2,4,6-triethylbenzene (5).** Yield: 92%; mp: 89–90 °C; ^1^H NMR (400 MHz, CDCl_3_, 0.9 mM): δ = 8.00 (s, 2H), 7.68 (d, *J* = 7.8 Hz, 2H), 7.35 (d, *J* = 8.0, 2H), 7.16–7.20 (m, 4H), 7.08–7.12 (m, 2H), 6.30 (s, 1H), 6.00 (s, 1H), 4.28 (d, *J* = 4.2 Hz, 2H), 4.09 (br. s, 1H), 4.08 (s, 4H), 3.75 (s, 4H), 2.66 (m, 6H), 2.33 (s, 3H), 2.20 (s, 3H), 1.09 (t, *J* = 7.5 Hz, 6H), 1.06 (t, *J* = 7.5 Hz, 3H) ppm; ^13^C NMR (100 MHz, CDCl_3_): δ = 158.28, 156.64, 148.55, 142.87, 142.49, 136.37, 134.51, 132.41, 127.16, 122.48, 122.02, 119.46, 119.00, 115.21, 113.60, 111.03, 103.55, 47.28, 45.51, 40.59, 24.20, 22.59, 22.52, 21.05, 16.77 ppm; HR-MS (ESI) calcd for C_40_H_49_N_6_ [M + H]^+^: 613.4018, found: 613.4012; *R_f_* = 0.12 [CHCl_3_/CH_3_OH (incl. 1% 7 M NH_3_ in CH_3_OH) 3:1 *v*/*v*].
